# Supplementary material for: Development of a method for Making Optimal Decisions for Intervention Flexibility during Implementation (MODIFI): a modified Delphi study
Source: Implement Sci Commun. 2024 Jun 17;5:64. doi: 10.1186/s43058-024-00592-x (PMC11181660; doi:10.1186/s43058-024-00592-x)
Supplement: Supplementary file 1 — Additional file 1. MODIFI: Making Optimal Decisions for Intervention Flexibility during Implementation. The final MODIFI method, including a description of MODIFI, definitions of key terms, necessary prerequisites, and a detailed step-by-step guide. [file 43058_2024_592_MOESM1_ESM.pdf]

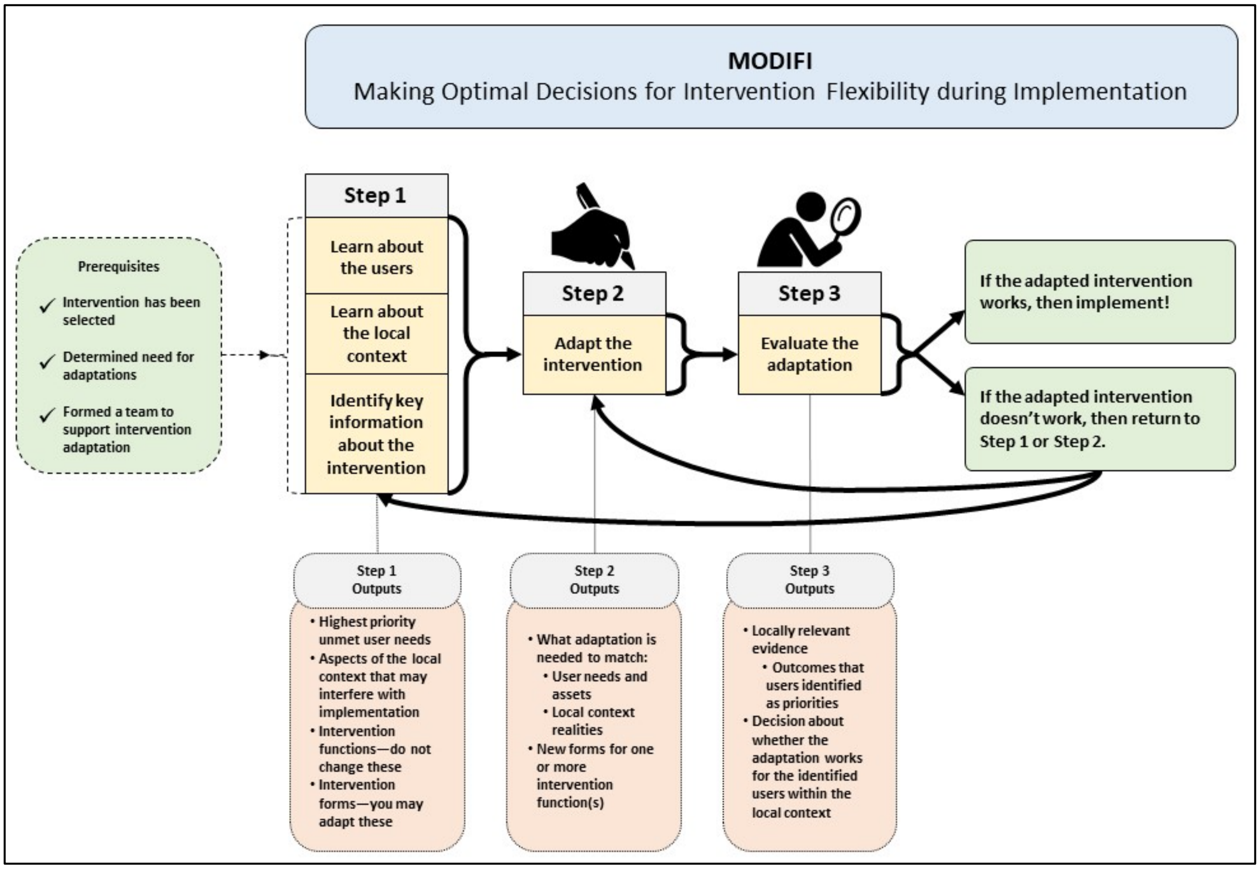

MODIFI Introduction

Overview

- MODIFI is a method for **adapting psychosocial intervention and prevention programs**
  - Some intervention/prevention programs are considered evidence-based practices or programs (EBPs), because there is research supporting their effectiveness
  - For simplicity, the term "intervention" is used throughout MODIFI

- MODIFI **integrates** the strengths of:
  - intervention development
  - implementation science
  - human centered design
  - cultural adaptation
  - participatory action research

- MODIFI is for making adaptations to **part of an intervention**—not a whole-intervention redesign
    - If you want to make multiple adaptations, it is recommended to complete the MODIFI steps for each adaptation (either one after another or at the same time in separate processes)
    - If you must make multiple adaptations using MODIFI all at once, a consequence might be that after completing MODIFI, you won't know which of those adaptations worked or didn't work—so this is not necessarily recommended

- MODIFI is intended to be a **feasible** method for **providers** or other **intervention implementers** / **decision-makers** working in applied settings
    - MODIFI is both **practical** and **rigorous**
    - MODIFI can be completed quickly—either **before or during** intervention implementation

- MODIFI's techniques can be carried out in a **range of ways**—to match the needs and resources of local settings, there are no prescribed numbers of participants, numbers of data collections, or time periods for data collections
    - MODIFI is a method that provides both **structure and flexibility**, because it is designed to be useful for a range of people working in a range of settings

- MODIFI should be completed in a **participatory, culturally responsive** way. The overarching principles of this approach include:
    - Partnering with relevant stakeholders
    - Valuing local knowledge/expertise
    - Synthesizing diverse perspectives
    - Collaborative problem solving
    - Checking for understanding

- Through the method of co-design, you are more likely to develop adaptations that work well for the users in the local context
    - Meeting their needs
    - Engaging their strengths

- Co-design involves **collaborating with users**
    - Different users/stakeholders can engage in this work in different ways

    - Throughout the adaptation process, the same users/stakeholders can be involved multiple times in different ways
- When deciding which forms of engagement are most appropriate for each user/stakeholder, consider which pieces of information each user/stakeholder has knowledge about (e.g., problem definition, real-world constraints, user/context strengths, possible solutions)

Definitions

- **Adaptation** = intervention modification that is planned in advance to suit the unique needs and characteristics of the implementation context and population of focus

- **Intervention functions** = the ways an intervention achieves its effects (intervention mechanisms, theory of change)
    - Core functions should not be adapted, as they are what makes the intervention effective
        - *For example, in behavioral activation, a core function is to increase contact with positive, reinforcing experiences*
- **Intervention forms** = the intervention activities that carry out its functions
    - Each intervention function takes one or more forms—a single function can take multiple forms
    - Intervention forms can be adapted to work better for new users and/or contexts
        - *In the example above, the function "increase contact with positive, reinforcing experiences" can take a number of forms—it can look a number of ways (e.g., daily activity monitoring, pleasant activity scheduling, rehearsal of assigned behaviors)*
- **Function/form table** = a table in which intervention functions and intervention forms are identified—each function is linked to the form(s) it takes (example function/form table is on next page).
    - MODIFI uses a function/form table to identify functions and forms prior to intervention adaptation.

Example Function/Form Table for Behavioral Activation		
**Problems**	**Functions**	**Forms**
• Depressed mood (feeling sad, empty, or hopeless)<br>• Reduced participation in activities of daily living	• Increase contact with positive, reinforcing experiences	• Daily activity monitoring<br>• Pleasant activity scheduling<br>• Cognitive rehearsal of assigned behaviors<br>• Role playing<br>• Skills training for obtaining & maintaining contact with positive reinforcement<br>• Functional analysis of behavior<br>• Individualized goal setting<br>• Stimulus control & contingency management procedures
• Increased avoidance behavior (avoiding activities that might cause discomfort or distress)	• Decrease escape/avoidance behavior<br>• Increase approach behavior	• Assess & identify avoidance patterns<br>• Identify alternate coping behaviors<br>• Skills training for tolerance of negative affect<br>• Functional analysis of behavior<br>• Individualized problem solving

- **MODIFI outcomes** = variables that you aim to improve by adapting the intervention
  - *For example, fit/appropriateness, acceptability, cultural responsiveness, usability, feasibility*
- **Implementation outcomes** = variables that show whether or not an intervention can be successfully implemented by local providers in the local context with the population of focus
  - *For example, cost, adoption, sustainment, fidelity*
- **Intervention outcomes** = variables that show whether or not an intervention can improve experiences for recipients (e.g., clinical/wellness/service outcomes)
  - *For example, anxiety, depression, life functioning*

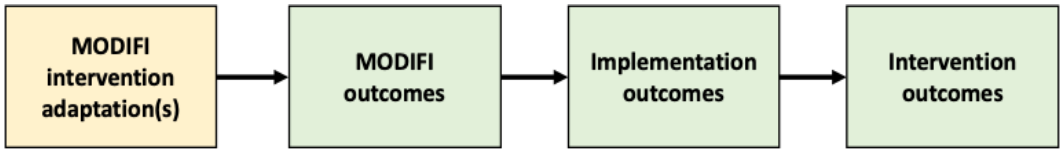

- **Human centered design** = an approach to designing an intervention that's based on information about the people and settings with which the intervention will be used—with the goal of making the intervention usable and engaging for the intended users
  - A solution-focused, action-oriented approach to solving problems
  - Based on understanding the needs, desires, preferences, values, and experiences of the people who will use an intervention
- **Primary users** = people who interact directly with the intervention, and whose needs are explicitly prioritized
  - *For example, primary users of an intervention are typically the providers and service recipients for whom the intervention is most intended*
- **Secondary users** = people who interact indirectly with the intervention or are not its primary focus, and whose needs can be addressed as long as this does not interfere with addressing the needs of primary users
  - *For example, secondary users of an intervention are organization leaders, service recipients' family members, etc.*

Prerequisites

- MODIFI is a method for intervention adaptation that can be used after a few preceding steps have been completed. . .
  - You have already selected an intervention to implement that addresses a problem for the population of focus, that relevant stakeholders believe is (or has the potential to be) appropriate, and that ideally has evidence for its effectiveness
  - You have determined that it is necessary to adapt that intervention for successful implementation—the original intervention cannot be implemented successfully due to problems with MODIFI outcomes (e.g., fit/appropriateness, usability, cultural responsiveness) and/or implementation outcomes (e.g., cost)
  - You have formed a team to support intervention adaptation
    - Adaptation teams work best when they contain a mix of people in different roles; specifically, primary users, people with expertise in the intervention (or the topic/problem it addresses), and people with expertise in intervention adaptation methods
    - When this is infeasible, MODIFI may be used by an individual intervention implementer / decision-maker

MODIFI

Step 1

Learn about the **users** and **context**—take an applied anthropological approach—listen, observe, and understand experiences within the local context

Identify the intervention's **functions** and **forms**—so you can adapt it while keeping it effective

Learn about the users

1. Specify who will be the "user(s)" of the adapted intervention (e.g., providers, service recipients)—this is who you're adapting for

- *NOTE: Multiple users can be considered simultaneously—integrate the information about their needs*
  - *Those applying MODIFI are likely primary or secondary users themselves*

2. Interview users—ask about their needs and assets related to the intervention and the topic/problem it addresses
   a. Ask users to rank their unmet needs in order of priority
   - *NOTE: When user needs are in contrast within or across user groups, needs should be prioritized in order of*

Learn about the local context

1. Identify which factors of the context's workflow, routines, personnel, decision-making, structure, etc. are most likely to impact the intervention's implementation
2. In the context where the adapted intervention will be implemented, conduct observations to capture information about the factors you identified, taking notes on exactly what you see and hear
   a. Observations can be conducted very efficiently
   b. Pick aspects of the context that are practical to observe (e.g., physical location, working hours, relevant services/people to shadow)
   c. Explain the purpose of your observation and

Identify key information about the intervention

To adapt an intervention while retaining/maximizing its effectiveness, you have to understand how it works

- Create a function/form table to map out how the intervention achieves its effects—this will show you what can and cannot be changed
- Often, core functions are not identified by intervention developers—use the steps below

1. Create a table with three columns: 1. Problems, 2. Functions, 3. Forms
2. Using the intervention materials (e.g., manual, website), consultation with intervention developers and/or experts, your professional experience, and the research literature, fill out the columns
   - *NOTE: All of these information sources may not be available to*

proximity to the intervention (e.g., primary users before secondary users)

3. If needed, use your professional experience and the research literature to elaborate upon what you learn from users

OUTPUT: Highest priority unmet user needs—these help inform intervention adaptations in Step 2

get permission from all involved

d. Remain unobtrusive, but know that you don't have to be invisible—be friendly, ask questions, reassure people that you are there to learn (not judge), and respect confidentiality

OUTPUT: Aspects of the local context that may interfere with intervention implementation—these help inform intervention adaptations in Step 2

you—use the best information available to fill out the function/form table

a. In column 1, list the problems the intervention aims to solve
b. In column 2, list the intervention's functions—the ways the intervention solves each problem
c. In column 3, list the form(s) that each function takes within the intervention (e.g., intervention activities)

With the function/form table completed, you will know how the intervention solves problems (the functions) and what forms each function takes

OUTPUT: Intervention functions—do not change these in Step 2, and intervention forms—you may adapt these in Step 2

- In the adapted intervention, make sure each function is represented in at least one form

Step 2

- This step uses a **co-design** method to adapt an intervention's **forms** while leaving functions intact

- Co-design involves **partnership between members of different groups** to explore challenging problems and identify solutions

How to co-design:

- **First,** identify who you want in your co-design sessions—human centered design works best with **diverse teams**
    - If possible, include at least one person with each of these roles: primary user, expert in the intervention (or the topic/problem it addresses), and expert in intervention adaptation methods
    - When this isn't feasible, consider which viewpoints may be absent from the team as you engage in co-design, and do your best to elevate those viewpoints as you present the information you gathered during Step 1

- **Then,** engage the group in **co-design sessions** (in-person or online), where you work as a team to:
    - **1. Understand the problem(s) to be solved through adaptation**
        - You present the information you gathered in Step 1 (e.g., highest priority unmet user needs, aspects of the local context that may interfere with intervention implementation, intervention functions)
        - Users/stakeholders present information about their experiences
    - **2. Generate possible solutions**
        - All co-design members contribute to brainstorming solutions to the problem(s) that you hope to solve through adaptation
        - During brainstorming, try to generate as many new ideas as possible—encourage openness and creativity, and defer judgment
        - After brainstorming a list of possible solutions, all co-design members decide collaboratively which solutions to select for co-creation (these are the intervention adaptations you will make)
    - **3. Co-create adaptations that solve the identified problem(s)**
        - All co-design members contribute to drafting intervention adaptation(s)
        - As you draft adaptation(s), make sure that each intervention function is represented in at least one form (refer to your function/form table from Step 1)
        - All co-design members contribute to iterating—co-create further adaptation drafts, building upon each version by asking yourselves, "What are the ways in which this solution could be just a little bit better?"
    - **4. Consider possible unintended consequences**

        - All co-design members contribute to considering the possible impacts of the drafted adaptation(s)
        - When considering the possible impacts of the drafted adaptation(s), discuss these questions:
            - Is this adaptation designed with specific goals in mind?
            - Is this adaptation aligned with intervention core functions?
            - Could there be unintended negative impacts of this adaptation (e.g., adoption, acceptability, appropriateness, cultural responsiveness, feasibility, cost, penetration, fidelity, sustainment, intervention outcomes)?
        - As a group, discuss any potential negative impacts, their likelihood, and their severity, then consider whether these can be mitigated with an implementation strategy and/or offset by positive impacts on other outcomes
    - **5. Continue to iterate until the adaptation is ready for evaluation**
        - Based on the findings of the impact analysis, further iteration may be warranted. In that case, co-create further adaptation drafts, building upon each version by asking yourselves, "What are the ways in which this solution could be just a little bit better?"
        - Finally, all co-design members reach group consensus by agreeing that the problem(s) have been solved well enough that the adaptation is ready for evaluation

OUTPUT: What adaptation is needed to match user needs / assets and local context realities

- New forms for one or more intervention function(s)

Step 3

- The goal of this step is to **generate evidence that is relevant to the identified users within the local context**
    - The goal is not to collect evidence that is generalizable to numerous other users and contexts—thus, efficient, feasible evaluation methods may be used

- Start by thinking about **what data you will need** to understand whether or not the intervention adaptation works for the identified users within the local context

    - If possible, include both quantitative and qualitative indicators of success (e.g., ratings of acceptability, quotes about cultural responsiveness, implementation outcomes, intervention outcomes)
    - Ultimately, make decisions about what data you will collect based on **what's feasible for you** in your context, alongside what you learned from the users/context in Step 1 about the **most important outcomes** to maximize during intervention adaptation

- Decide **how you will measure the outcomes** you've chosen, **how often** you will collect data, and **what you will need to see** in order to conclude that the adaptation works for the identified users within the local context
    - These decisions are also based on what you can actually track in your context—data collection may be as narrow as a provider asking a service recipient if the adaptation is acceptable to them at each session while the adaptation is implemented—or it may be as complex as collecting data on multiple outcomes with multiple users over time before the adaptation is implemented

What's Next?

If the adapted intervention works for the identified users within the local context. . .

> . . . implement the adapted intervention!
>
> . . . if resources allow, implement while collecting additional data (e.g., on the outcomes you've chosen, and/or on additional changes that occur during implementation)

If the adapted intervention does not work for the identified users within the local context (or a subset of the identified users). . .

> . . . return to Step 1 if you need to learn more about the users, context, and/or intervention before further adaptation, then continue from there
>
> . . . or return to Step 2 if you know what further adaptation is needed, then continue from there
