# Supplementary material for: Development of a method for Making Optimal Decisions for Intervention Flexibility during Implementation (MODIFI): a modified Delphi study
Source: Implement Sci Commun. 2024 Jun 17;5:64. doi: 10.1186/s43058-024-00592-x (PMC11181660; doi:10.1186/s43058-024-00592-x)
Supplement: Supplementary file 2 — Additional file 2. Summary of original MODIFI components and revision decisions based on Round 1 results. Detailed descriptions of revisions made to each MODIFI component as informed by Round 1 results. [file 43058_2024_592_MOESM2_ESM.pdf]

Summary of original MODIFI components and revision decisions based on Round 1 results

Original MODIFI component	Revision decision based on Round 1 results	How the component was revised
Phase 1. Decide what needs adaptation		
Phase 1a. Specify for whom it's crucial to adapt the identified EBP or component.<br>Step 1. Specify who will be the "users" of the adapted EBP.<br>Step 2. Identify the users' needs based on the perspectives of users themselves.<br>Step 3. Rank the users' needs in order of priority.	Revised	· Clarified that the "users" are the people who you're adapting for<br>· Added how to consider the needs of multiple user groups simultaneously<br>· Added to consider user assets in addition to needs<br>· Changed the priority ranking to be done by the users instead of the EBP adapter<br>· Added how to proceed when user needs are in contrast within or across user groups<br>· De-emphasized the use of professional experience and research literature to elevate the importance of user voice<br>· Added the output that will be achieved following these steps
Phase 1b. Identify which outcome(s) you hope to maximize through EBP adaptation.	Removed	· Added an "Introduction" component that includes definitions of clinical/service outcomes and implementation outcomes<br>· Added a new component to identify intervention functions to retain during adaptation
Phase 2. Adapt the EBP		
Phase 2a. Identify the EBP mechanisms of change (the ways the EBP achieves its intended clinical impacts).<br>Step 1a. Interview the people involved in the development and initial testing of the EBP to ask about its mechanisms of change.<br>Step 1b. Find key themes in the responses regarding the EBP mechanisms of change.<br>Step 2. Identify hypothesized EBP mechanisms of change.<br>Step 3. Compare hypothesized EBP mechanisms of change to relevant research literature. If your hypotheses are not supported, revise hypothesized	Revised	· Instead of EBP mechanisms of change, added a new component to identify intervention functions to retain during adaptation<br>· Instead of primarily relying on interviews with intervention developers/researchers, added steps for creating a function/form table based on multiple available information sources<br>· Removed the need for infeasible qualitative coding methods

EBP mechanisms of change based on the literature.		
Phase 2b. Adapt EBP component(s) to maximize your targeted outcome(s) while leaving EBP mechanisms intact.<br>Step 1. Discover information about the context and the product using HCD methods.<br>Step 2. Design and build adapted EBP component(s) using HCD methods.	Revised	· Instead of selecting among a lengthy menu of HCD methods, added steps for using co-design to collaboratively develop intervention adaptations<br>· Added information regarding the participatory nature of co-design<br>· Added information regarding how to follow the co-design method
Phase 3. Pilot test the adapted EBP		
Phase 3. Pilot test the adapted EBP component(s) and ensure that the EBP mechanisms are intact.<br>Step 1. Choose identified user(s) to include in the pilot test.<br>Step 2. Choose which adapted EBP component(s) to include in the pilot test.<br>Step 3a. During the pilot test, collect pre-post data on your targeted outcome(s) and EBP mechanisms.<br>Step 3b. During the pilot test, track information about characteristics of the users (e.g., provider, client) and the context.	Revised	· Instead of conducting a pilot test, added steps for generating locally relevant evidence<br>· Removed the need for infeasible pilot study methods<br>· Clarified how to gather information that will show whether or not the adaptation works for the intended users and context<br>· Added a "What's Next?" component that explains how to move forward and/or repeat MODIFI steps based on the results
