# Supplementary material for: Development of a method for Making Optimal Decisions for Intervention Flexibility during Implementation (MODIFI): a modified Delphi study
Source: Implement Sci Commun. 2024 Jun 17;5:64. doi: 10.1186/s43058-024-00592-x (PMC11181660; doi:10.1186/s43058-024-00592-x)
Supplement: Supplementary file 3 — Additional file 3. Summary of revised MODIFI components and final revision decisions based on Round 2 results. Detailed descriptions of final revisions made to each MODIFI component as informed by Round 2 results. [file 43058_2024_592_MOESM3_ESM.pdf]

Summary of revised MODIFI components and final revision decisions based on Round 2 results

Revised MODIFI component	Final revision decision based on Round 2 results	How the component was revised
MODIFI Introduction		
Overview	Revised	· Moved content from later in MODIFI to this section—the content describing the participatory, culturally responsive nature of MODIFI
Definitions	Revised	· Added a definition of adaptation<br>· Used a different example when defining intervention function and intervention form<br>· Added an example intervention function/form table<br>· Reorganized outcomes into clearer sections
Prerequisites	Retained	· N/A
MODIFI Step 1		
Learn about the users	Revised	· Integrated more participatory language<br>· Added that people applying MODIFI likely are primary or secondary users themselves
Learn about the local context	Retained	· N/A
Identify key information about the intervention	Revised	· Added that often core functions are not identified by intervention developers<br>· Added examples of intervention materials to reference<br>· Added the suggestion to reach out to developers when possible
MODIFI Step 2		
Co-design description	Revised	· Moved content from this section to the MODIFI Introduction—the content describing the participatory, culturally responsive nature of MODIFI

How to co-design	Revised	· Added steps for how to consider possible unintended consequences<br>· Added steps for how to continue to iterate until the adaptation is ready for evaluation
MODIFI Step 3		
Testing whether or not the adaptation works	Retained	· N/A
What’s Next?		
Next steps	Revised	· Added the recommendation for further evaluation and iteration if resources allow
