# Supplementary material for: Development of a method for Making Optimal Decisions for Intervention Flexibility during Implementation (MODIFI): a modified Delphi study
Source: Implement Sci Commun. 2024 Jun 17;5:64. doi: 10.1186/s43058-024-00592-x (PMC11181660; doi:10.1186/s43058-024-00592-x)
Supplement: Supplementary file 4 — Additional file 4. MODIFI Case Example. Case example of the final MODIFI method used by school-based mental health care providers to adapt an evidence-based intervention. [file 43058_2024_592_MOESM4_ESM.pdf]

MODIFI Case Example

An elementary school decides to implement Trauma-Focused Cognitive-Behavioral Therapy (TF-CBT; Cohen et al., 2017), an evidence-based intervention for child and adolescent trauma, to best meet the mental health needs of their students. The school's mental health care providers (SMHP) identify that the session sequence and content of TF-CBT requires adaptation for it to be adequately implemented in their school. The SMHPs recognize they do not have access to people with expertise in adaptation methods. As such, they form an adaptation team that includes a lead SMHP and two other SMHPs in the school.

MODIFI Step 1: Learn About the Users

The adaptation team identifies SMHPs (TF-CBT implementers) and students (TF-CBT recipients) as primary users of the intervention; teachers and parents of children receiving TF-CBT are identified as secondary users. The adaptation team recognizes their limited resources (e.g., time) and prioritizes information from primary users. The lead SMHP first interviews the other SMHPs at the school and reflects on the interview questions themself to determine what are the needs to successfully implement TF-CBT over the coming year and what assets currently exist to support implementation. From these interviews, the adaptation team learns that their school has protected space to deliver TF-CBT and several providers trained in TF-CBT to meet student need. They also learn that they need (a) the session length to be flexible to deliver TF-CBT during the school day, (b) protected time and space for students to decompress post-session, and (c) more decision latitude around how much time to spend in each step of TF-CBT before moving onto the next. The team rank-orders these needs as follows: a, c, b.

MODIFI Step 1: Learn About the Local Context

To learn more about the context in which TF-CBT will be implemented, the lead SMHP conducts morning observations for two of their colleagues. The goal of these observations is to gather information about what their colleagues do, who they see and when, where they see students, what are students' transitions, and any other structural or process information that would be useful in identifying barriers to implementing TF-CBT. The SMHP learns that students receiving TF-CBT are generally seen prior to 12pm in SMHPs' private offices. The transitions depended on the bell schedule. Some students transition away from and back into classroom instruction, whereas others transition from classroom instruction to a shared activity like recess. Observations also reveal that the amount of instructional time students miss depends on TF-CBT session length and the intensity of session content. In one instance following a session involving intense emotional content, a student required more time to regulate their emotions before re-entering instructional time than was originally planned.

MODIFI Step 1: Identify Key Information About the Intervention

The SMHPs collaboratively create a function/form table (see Table 1). They first list the problems that TF-CBT addresses (e.g., posttraumatic symptoms) and then brainstorm what functions TF-CBT accomplishes (e.g., change trauma-related cognitions). Finally, the team identifies forms that each function takes in TF-CBT (e.g., cognitive coping skills).

MODIFI Step 2: Adapt the Intervention

The lead SMHP recruits a team to co-design TF-CBT adaptations. They do not have access to experts in TF-CBT or intervention adaptation but believe their team includes enough expertise in TF-CBT to proceed, and they reference a TF-CBT manual and pedagogical articles on intervention adaptation throughout the co-design.

To diversify the team, the lead SMHP invites two teachers (secondary users) who have students in their classrooms receiving TF-CBT. The team of three SMHPs and two teachers meet to work through the five components outlined above. The Example Table below presents the problem(s) to be solved, proposed solutions, adaptations, and unintended consequences as identified by the co-design team. The problems are identified using information from Step 1, specifically, user assets and needs and aspects of the local context that may interfere with intervention implementation.

The team identifies two possible solutions (Example Table 1a and 1b) but believe the potential unintended consequences would either decrease the effectiveness of TF-CBT and/or result in the same level of disruption to students' schedules. The team agrees that solution 1c is the most feasible as it addresses the needs of SMHPs (shorter and more flexible sessions) and students (time to decompress, less instructional time lost). The team discusses the possible unintended consequence of solution 1c (inadequate time for all TF-CBT components) and references their function/form table to ensure that all functions can be accomplished by the TF-CBT activities completed early during the course of treatment (e.g., cognitive coping skills change trauma-related cognitions).

Example Table for Iterative Co-Design of TF-CBT Adaptations

Problem(s) to be Solved	Possible Solution		Adaptations to Solve Problem	Unintended Consequences
The prescribed length of TF-CBT sessions (50-90 minutes) is too long to fit into the schedules of SMHP serving multiple students and is causing a substantial loss of instructional time for students.<br><br>Students are having trouble joining their classrooms after emotionally charged sessions.<br><br>SMHP are struggling to get to and through the trauma narration with all students because of available time, comfort level with trauma narration, and/or because the student is requiring more time to move through the prior TF-CBT components.	1a.	Have more frequent, but briefer sessions.<br><br>Build in time for students to decompress post-session.	Schedule students for 3-4 20-minute sessions every week.<br><br>Block 10-15 minutes post session for decompression time should the student require it.	The proposed session length (20min) is too short a time to effectively deliver components of TB-CBT and particularly those components SMHP are already struggling to complete (e.g., trauma narration).<br><br>Students' schedules are disrupted multiple times a week as opposed to once. Further, if each session is emotionally charged, the student will need decompressing time multiple days a week, leading to decreased access to instructional time.
	1b.	Have more frequent, but briefer sessions where the session length is dependent on component of TF-CBT being engaged with.<br><br>Build in time for students to decompress post-session.	Schedule students for different times based on TF-CBT component engaged in (e.g., 30 min weekly for psychoeducation or relaxation skills, and two 30 min weekly sessions for the trauma narration).<br><br>Block 10-15 minutes post session for decompression time should the student require it.	SMHP incentivized to stay in TF-CBT components prior to trauma narration to limit the total weekly session length, which is time needed to serve other students.<br><br>Students' schedules are disrupted multiple times a week and for longer amounts of time as the student moves further into TF-CBT leading to decreased access to instructional time.
	1c.	Have briefer sessions where the session length is dependent on the component of TF-CBT.<br><br>Create expectation for SMHP to get through cognitive coping and processing skills (component 5 of TF-CBT) but build in flexibility around the completion of trauma narration (component 6) and all components thereafter.<br><br>Build in time for students to decompress post-session.	Schedule students for 30 min weekly sessions, spreading content across multiple sessions if needed. One 60-minute session the first time the student engages in the trauma narration.<br><br>To ensure forms remain that serve each function (see function and form table from Step 1-3), SMHP are expected to get through component 5.<br><br>Block 10-15 minutes post session for decompression time should the student require it.	May not make it through all TF-CBT components, which may decrease the positive impact of TF-CBT.

MODIFI Step 3: Evaluate the Adaptation

The co-design team determines what evidence they should collect to assess whether the adaptations are working for them and their students. Teachers agree to keep notes and check in with the SMHPs about how students are reintegrating into their classrooms after TF-CBT sessions and how much instructional time is being missed. The SMHPs similarly agree to keep notes about how the shorter session lengths are going, what components of TF-CBT they are able to complete (a feasible fidelity check for their team), how often students use the decompression time, and whether that length of time is appropriate. The SMHPs also have access to data from their sessions to determine whether the intended impact of TF-CBT on student functioning is maintained after adaptation. The lead SMHP decides to schedule half-day observations twice per year to gather information

related to the user needs and assets that were identified in Step 1. The SMHPs and teachers also complete a measure of acceptability twice per year. The team schedules time to analyze and discuss these data twice per year: once mid-year to evaluate progress and once at the end of the year to understand what worked well and what, if any, other adaptations might be helpful moving forward. After reviewing data at the end of the year, the team agrees that the TF-CBT adaptations had their intended effects and decide to use the MODIFI process during the upcoming academic year to tackle the problem of not enough SMHPs getting to and through the trauma narrative.
