# Supplementary material for: Development of a method for Making Optimal Decisions for Intervention Flexibility during Implementation (MODIFI): a modified Delphi study
Source: Implement Sci Commun. 2024 Jun 17;5:64. doi: 10.1186/s43058-024-00592-x (PMC11181660; doi:10.1186/s43058-024-00592-x)
Supplement: Supplementary file 5 — Additional file 5. Standards for Reporting Qualitative Research (SRQR). Completed checklist for qualitative research reporting standards. [file 43058_2024_592_MOESM5_ESM.pdf]

Standards for Reporting Qualitative Research (SRQR)

No.	Topic	Item Location in Manuscript (page numbers)
S1	Title	1
S2	Abstract	2
S3	Problem formulation	3-6
S4	Purpose or research question	7
S5	Qualitative approach and research paradigm	7-9
S6	Researcher characteristics and reflexivity	7-9
S7	Context	7-9
S8	Sampling strategy	7-9
S9	Ethical issues pertaining to human subjects	9
S10	Data collection methods	9-11
S11	Data collection instruments and technologies	9-11
S12	Units of study	9-11
S13	Data processing	9-11
S14	Data analysis	9-11
S15	Techniques to enhance trustworthiness	9-11
S16	Synthesis and interpretation	12-25, Additional File 1
S17	Links to empirical data	Figure 3, Figure 4
S18	Integration with prior work, implications, transferability, and contribution(s) to the field	25-27
S19	Limitations	27
S20	Conflicts of interest	30
S21	Funding	30
